# Housing, Neighborhood Factors, and Cognitive Status of Older Adults Living in Houses Versus Apartments

**DOI:** 10.1093/geroni/igab046.2368

**Published:** 2021-12-17

**Authors:** Ethan Siu Leung Cheung, Ada Mui

**Affiliations:** 1 Columbia University, New York, New York, United States; 2 Columbia University, COLUMBIA UNIVERSITY, New York, United States

## Abstract

This study examines associations between housing, neighborhood factors and cognitive status among community-dwelling older adults, and how the associations differ between older adults who live in houses and in apartment buildings. Specifically, using the neighborhood stressor theory, three research questions are examined: 1) What individual-level factors predict late-life cognitive status? 2) After controlling for individual-level factors, what housing and neighborhood factors are significant in predicting older adults’ cognitive status? 3) How do individual, housing, neighborhood predictors of cognitive status differ between house and apartment residents? Using data from the Wave 3 NSHAP, multilevel linear regression analyses are conducted with the total sample. Results suggest that individual-level factors including young-age, female, white, and having a bachelor’s degree are associated with better cognitive status. After controlling for individual-level factors, housing and neighborhood factors including quality maintenance and high level of community safety are associated with higher cognitive scores. In addition to the additive model, we also test the interactive effect between housing type and three level of factors –individual level, housing, and neighborhood factors. Findings suggest that the joint effect of depression and housing type on cognitive status is significant. To explore the last research question, we conduct parallel regression analyses by housing type. Findings suggest that quality maintenance and high level of community safety are associated with higher cognitive scores among house residents only. Findings highlight the predictors of cognitive health vary between older adults living in different residential environments.

